# 525 Tiered Orientation Model for Nurses in a Burn Unit

**DOI:** 10.1093/jbcr/irad045.122

**Published:** 2023-05-15

**Authors:** Natalie Fitzgerald, Cynthia Julian, Shawna Thomas

**Affiliations:** Richard M. Fairbanks Burn Center at Eskenazi Health, Indianapolis, Indiana; Richard M. Fairbanks Burn Center at Eskenazi Health, Indianapolis, Indiana; Richard M. Fairbanks Burn Center at Eskenazi Health, Indianapolis, Indiana; Richard M. Fairbanks Burn Center at Eskenazi Health, Indianapolis, Indiana; Richard M. Fairbanks Burn Center at Eskenazi Health, Indianapolis, Indiana; Richard M. Fairbanks Burn Center at Eskenazi Health, Indianapolis, Indiana; Richard M. Fairbanks Burn Center at Eskenazi Health, Indianapolis, Indiana; Richard M. Fairbanks Burn Center at Eskenazi Health, Indianapolis, Indiana; Richard M. Fairbanks Burn Center at Eskenazi Health, Indianapolis, Indiana

## Abstract

**Introduction:**

As an acuity adaptable unit is is sometimes difficult to get nurses through an orientation in a manner that doesn't scare them off but still covers all their educational needs. The number of fluid resuscitations seen is approximately 30 per year which means that when one occurs new nursing staff needs to have exposure. However, it is a difficult experience to work through a large ICU admission as a new nurse even with preceptor support. Due to these issues the unit adopted a tiered orientation process. This process set forth task blocks as a focus during orientation versus entire patient care. For example, the new nurse and preceptor assignment could be the new fluid resuscitation in week one of orientation. However, the new nurse would only work on documentation of patient care. The preceptor would be responsible for procedures, dressing changes, fluid, and pharmacologic management that goes into caring for the acute burn. the new nurse could also be a part of these processes but that is not their focus during Tier 1. In Tier 2 the new nurse would start providing more patient care in addition to their documentation, the preceptor would still perform ICU level care items. In the final tier the orientee would do all direct care of the patient including documenation. This tiered approach allowed nurses to experience all levels of patient care without overwhelming them.

**Methods:**

The process was brought to the shift coordinators to give them all a sense of how staffing assignments would be made during orientation. Education was presented to preceptors at unit council meetings and then in 1 on 1 meetings as needed. Additional, touch base meetings occurred every other week during the start of this process starting in May of 2022. During this time 7 new nurses were on-boarded. 7 unique preceptors were utilized. New hires were met with weekly on an informal basis from weeks 1-5 by either the clinical educator or the nurse manager. Each new nurse and their preceptor had formal meetings with the nurse manager, clinical educator or both at 6, 10 and 12 weeks. A short survey was sent to all preceptors following the initial 5 months of this new process.

**Results:**

50% of all preceptors found this new model to be an improvement over the previous model. 33% did not agree or disagree on if the process was an improvement. 2 out of the 7 preceptors had not precepted before which might account for the 33%. 40% of the preceptors felt satisfied with the overall process for orientation. 83% of the preceptors felt that they had adequate resources to orient new nurses to the unit.

**Conclusions:**

It appears that the adoption of a tiered process worked well during the initial evaluation phase. However, the n was small and additional evaluation would be beneficial to further address how impactful a tiered orientation process is for on-boarding new nursing staff.

**Applicability of Research to Practice:**

It important for nursing leaders to evaluate and update how to best meet the needs of staff to create competency and safety.

